# Supplementary material for: Optimizing crop varietal mixtures for viral disease management: A case study on cassava virus epidemics
Source: PLoS Comput Biol. 2025 Sep 18;21(9):e1012842. doi: 10.1371/journal.pcbi.1012842 (PMC12469245; doi:10.1371/journal.pcbi.1012842)
Supplement: S1 Appendix — Mathematical derivations and the full model equations. (PDF) [file pcbi.1012842.s001.pdf]

Israël Tankam Chedjou, Ruairí Donnelly, Christopher A. Gilligan "Optimizing crop varietal mixtures for viral disease management: A case study on cassava virus epidemics"

Supporting information contains full mathematical derivations, generalisations, and illustrative outputs that complement the main text. S1 Appendix provides modelling details, including the complete system of equations and derivations. S2 Appendix presents the analytical expression for the basic reproduction number together with a short epidemiological interpretation. S3 Appendix generalises the framework to an arbitrary number of crop varieties, giving the transition matrix, its inverse, and feeding proxies for mixtures of size n. S4 Appendix illustrates CropMix functionality through example screenshots, baseline versus optimal mixture visualisations, and viruliferous vector dynamics. Finally, S5 Appendix explores the impact of roguing on optimal resistant–susceptible mixtures under cassava mosaic disease, showing that the susceptible monoculture remains the optimal configuration across all scenarios.

S1 Appendix, Modelling details

The efficiency of transmission increases with longer feeding times and multiple infected whiteflies, so we need a modelling framework that can capture the lifespan of insect vectors on different plant types: S_A, S_B, L_A, L_B, I_A and I_B.

We name ρ_J^X (make the difference with the roguing rate ρ) the probability that a viruliferous vector that acquired the pathogen on a variety X ($X = A$ or B) is found feeding on a type J plant ($J \in \{S_A, S_B, L_A, L_B, I_A, I_B\}$). It measures the proportion of its lifespan that a vector that acquired the virus on a variety X spends of a plant of type J. The model reads:

$$\begin{cases} \dot{S}_A = -\beta^A(\rho_{S_A}^A V^A + \rho_{S_A}^B V^B) + \rho d_A I_A \\ \dot{L}_A = \beta^A(\rho_{S_A}^A V^A + \rho_{S_A}^B V^B) - \gamma_A L_A \\ \dot{I}_A = \gamma_A L_A - \rho d_A I_A \\ \dot{S}_B = -\beta^B(\rho_{S_B}^A V^A + \rho_{S_B}^B V^B) + \rho d_B I_B \\ \dot{L}_B = \beta^B(\rho_{S_B}^A V^A + \rho_{S_B}^B V^B) - \gamma_B L_B \\ \dot{I}_B = \gamma_B L_B - \rho d_B I_B \\ \dot{V}^A = \alpha^A \left(I_A \dfrac{F}{K} - V^A \rho_{I_A}^A - V^B \rho_{I_A}^B \right) - (r+\omega) V^A \\ \dot{V}^B = \alpha^B \left(I_B \dfrac{F}{K} - V^A \rho_{I_B}^A - V^B \rho_{I_B}^B \right) - (r+\omega) V^B. \end{cases} \tag{S1.1}$$

- First proxy

  Let $\vec{\rho}^A = (\rho_{S_A}^A, \rho_{L_A}^A, \rho_{I_A}^A, \rho_{S_B}^A, \rho_{L_B}^A, \rho_{I_B}^A)$ the vector of all the probabilities of all the feeding locations of vectors that acquired the virus on variety A. Under the assumption that viruliferous vectors V^A are born on I_A, it obeys the equation whose solution is the relative value of the vector $M^{-1}{\rho_0^A}^T$, where ${\rho_0^A}^T = (0,0,1,0,0,0)$, and M is the matrix formed by the rates of transition of vector locations detailed a bit further (this is a consequence of the relation $\int_0^\infty \vec{\rho}^A(t)dt = M^{-1}{\rho_0^A}^T$ for linear Markov-chains, see expected lifspan proportions in [Donnelly et al., 2015]). We obtain:

$$\vec{\rho}^A = \frac{1}{\omega + r + \sigma}\left(\sigma\frac{S_A}{K}, \sigma\frac{L_A}{K}, \sigma\frac{I_A}{K} + \omega + r, \sigma\frac{S_B}{K}, \sigma\frac{L_B}{K}, \sigma\frac{I_B}{K}\right).$$

- Second proxy

Let $\vec{\rho}^B = (\rho^B_{S_A}, \rho^B_{L_A}, \rho^B_{I_A}, \rho^B_{S_B}, \rho^B_{L_B}, \rho^B_{I_B})$ the vector of all the probabilities of all the feeding locations of vectors that acquired the virus on variety B. Under the assumption that viruliferous vectors V^B are born on I_B, $\vec{\rho}^B$ obeys the equation whose solution is the relative value of the vector $M^{-1}{\rho_0^B}^T$, where ${\rho_0^B}^T = (0,0,0,0,0,1)$. We obtain

$$\vec{\rho}^B = \frac{1}{\omega + r + \sigma}\left(\sigma\frac{S_A}{K}, \sigma\frac{L_A}{K}, \sigma\frac{I_A}{K}, \sigma\frac{S_B}{K}, \sigma\frac{L_B}{K}, \sigma\frac{I_B}{K} + \omega + r\right).$$

The transition matrix If we refer to the plant state variables S_A, S_B, L_A, L_B, I_A and I_B as X_i, with indices $i \in \{1, \ldots, 6\}$, then the entry M_{ij}, $i \neq j$ of the insect transition matrix is the rate at which infectious insects from X_i plants settle on type X_j plants, and the entry M_{jj} corresponds to the rate at which infected insects leave plants of type j. The entries M_{ij}, with $i \neq j$ obviously come from the insect dispersion applied to the considered class (σx_i), while infectious insects can be removed from X_j plants either by dispersing away $(\sigma(1 - x_i))$, dying (ω), or recovering from the disease (r). The transition matrix therefore reads:

$$M =$$

$$\begin{bmatrix} -[\omega + \sigma(1 - s_A) + r] & \sigma s_A & \sigma s_A & \sigma s_A & \sigma s_A & \sigma s_A \\ \sigma l_A & -[\omega + \sigma(1 - l_A) + r] & \sigma l_A & \sigma l_A & \sigma l_A & \sigma l_A \\ \sigma i_A & \sigma i_A & -[\omega + \sigma(1 - i_A) + r] & \sigma l_A & \sigma l_A & \sigma l_A \\ \sigma s_B & \sigma s_B & \sigma s_B & -[\omega + \sigma(1 - s_B) + r] & \sigma s_B & \sigma s_B \\ \sigma l_B & \sigma l_B & \sigma l_B & \sigma l_B & -[\omega + \sigma(1 - l_B) + r] & \sigma l_B \\ \sigma i_B & \sigma i_B & \sigma i_B & \sigma i_B & \sigma i_B & -[\omega + \sigma(1 - i_B) + r] \end{bmatrix}$$

With the notations $s_A = \frac{S_A}{K}, l_A = \frac{L_A}{K}, i_A = \frac{I_A}{K}, s_B = \frac{S_B}{K}, l_B = \frac{L_B}{K}, i_B = \frac{I_B}{K}$; and we have:

$$M^{-1} = \frac{1}{(\omega + r + \sigma)(\omega + r)} \begin{bmatrix} -\sigma s_A - r - \omega & -\sigma s_A & -\sigma s_A & -\sigma s_A & -\sigma s_A & -\sigma s_A \\ -\sigma l_A & -\sigma l_A - r - \omega & -\sigma l_A & -\sigma l_A & -\sigma l_A & -\sigma l_A \\ -\sigma i_A & -\sigma i_A & -\sigma i_A - r - \omega & -\sigma i_A & -\sigma i_A & -\sigma i_A \\ -\sigma s_B & -\sigma s_B & -\sigma s_B & -\sigma s_B - r - \omega & -\sigma s_B & -\sigma s_B \\ -\sigma l_B & -\sigma l_B & -\sigma l_B & -\sigma l_B & -\sigma l_B - r - \omega & -\sigma l_B \\ -\sigma i_B & -\sigma i_B & -\sigma i_B & -\sigma i_B & -\sigma i_B & -\sigma i_B - r - \omega \end{bmatrix}$$

Final equation From the above, if we recall that $S_A = \theta K - L_A - I_A$ and $S_B = (1-\theta)K - L_B - I_B$ we can drop the S_A and S_B equations from (S1.1), if we additionally consider the proportions $l_A = \frac{L_A}{K}, i_A = \frac{I_A}{K}, l_B = \frac{L_B}{K}$ and $i_B = \frac{I_B}{K}$, and the constant $\psi = \frac{\sigma}{\sigma + \omega + r}$, then the model in proportions of plant relative to the field density K reads:

$$\begin{cases} \dot{l}_A = \dfrac{\psi}{K}\beta^A(\theta - l_A - i_A)(V^A + V^B) - \gamma_A l_A \\ \dot{i}_A = \gamma_A l_A - \rho d_A i_A \\ \dot{l}_B = \dfrac{\psi}{K}\beta^B(1 - \theta - l_B - i_B)(V^A + V^B) - \gamma_B l_B \\ \dot{i}_B = \gamma_B l_B - \rho d_B i_B \\ \dot{V}^A = \alpha^A\left[i_A F - \psi i_A(V^A + V^B)\right] - (\omega + r)\left(1 + \alpha^A\dfrac{\psi}{\sigma}\right)V^A \\ \dot{V}^B = \alpha^B\left[i_B F - \psi i_B(V^A + V^B)\right] - (\omega + r)\left(1 + \alpha^B\dfrac{\psi}{\sigma}\right)V^B. \end{cases} \tag{S1.2}$$
