# Supplementary material for: Optimizing crop varietal mixtures for viral disease management: A case study on cassava virus epidemics
Source: PLoS Comput Biol. 2025 Sep 18;21(9):e1012842. doi: 10.1371/journal.pcbi.1012842 (PMC12469245; doi:10.1371/journal.pcbi.1012842)
Supplement: S2 Appendix — Analytical expression for the basic reproduction number and a short epidemiological interpretation. (PDF) [file pcbi.1012842.s002.pdf]

S2 Appendix, Basic reproduction number

Here we compute the basic reproduction number as in [Diekmann et al., 2009]. Let's consider the model equations (S1.2) in S1 Appendix. The linearized system around the disease-free equilibrium (0, 0, 0, 0, 0, 0) follows:

$$\begin{cases} \dot{l}_A = \dfrac{\psi}{K}\beta^A\theta(V^A + V^B) - \gamma_A l_A \\ \dot{i}_A = \gamma_A l_A - \rho d_A i_A \\ \dot{l}_B = \dfrac{\psi}{K}\beta^B(1-\theta)(V^A + V^B) - \gamma_B l_B \\ \dot{i}_B = \gamma_B l_B - \rho d_B i_B \\ \dot{V}^A = \alpha^A F i_A - (\omega + r)\left(1 + \alpha^A \dfrac{\psi}{\sigma}\right) V^A \\ \dot{V}^B = \alpha^B F i_B - (\omega + r)\left(1 + \alpha^B \dfrac{\psi}{\sigma}\right) V^B. \end{cases} \tag{S2.1}$$

We now rewrite the linearized system in the form:

$$\dot{X} = (T + \Sigma)X. \tag{S2.2}$$

The matrix T corresponds to the transmissions and the matrix Σ to transitions. All the epidemiological events that lead to infection are incorporated into the model via T, and all the other events via Σ. If we refer to the state variables with indices i and j , $i, j \in \{1, \dots, 6\}$, then the entry T_{ij} is the rate at which individuals in infected state j give rise to individuals in infected state i in the linearized system (S2.1) and Σ accounts for the remaining terms. We therefore have:

$$T = \begin{bmatrix} 0 & 0 & 0 & 0 & \frac{\theta\psi\beta^A}{K} & \frac{\theta\psi\beta^A}{K} \\ 0 & 0 & 0 & 0 & 0 & 0 \\ 0 & 0 & 0 & 0 & \frac{(1-\theta)\psi\beta^B}{K} & \frac{(1-\theta)\psi\beta^B}{K} \\ 0 & 0 & 0 & 0 & 0 & 0 \\ 0 & \alpha^A F & 0 & 0 & 0 & 0 \\ 0 & 0 & 0 & \alpha^B F & 0 & 0 \end{bmatrix}$$

and

$$\Sigma = \begin{bmatrix} -\gamma_A & 0 & 0 & 0 & 0 & 0 \\ \gamma_A & -\rho d_A & 0 & 0 & 0 & 0 \\ 0 & 0 & -\gamma_B & 0 & 0 & 0 \\ 0 & 0 & \gamma_B & -\rho d_B & 0 & 0 \\ 0 & 0 & 0 & 0 & -(\omega + r)\left(1 + \frac{\alpha^A\psi}{\sigma}\right) & 0 \\ 0 & 0 & 0 & 0 & 0 & -(\omega + r)\left(1 + \frac{\alpha^B\psi}{\sigma}\right) \end{bmatrix}$$

From which the next-generation matrix is:

$$-T\Sigma^{-1} = \begin{bmatrix} 0 & 0 & 0 & 0 & \frac{\theta\psi\beta^A\sigma}{K(\omega+r)(\alpha^A\psi+\sigma)} & \frac{\theta\psi\beta^A\sigma}{K(\omega+r)(\alpha^B\psi+\sigma)} \\ 0 & 0 & 0 & 0 & 0 & 0 \\ 0 & 0 & 0 & 0 & -\frac{(-1+\theta)\psi\beta^B\sigma}{K(\omega+r)(\alpha^A\psi+\sigma)} & -\frac{(-1+\theta)\psi\beta^B\sigma}{K(\omega+r)(\alpha^B\psi+\sigma)} \\ 0 & 0 & 0 & 0 & 0 & 0 \\ \frac{\alpha^A F}{\rho d_A} & \frac{\alpha^A F}{\rho d_A} & 0 & 0 & 0 & 0 \\ 0 & 0 & \frac{\alpha^B F}{\rho d_B} & \frac{\alpha^B F}{\rho d_B} & 0 & 0 \end{bmatrix}$$

Whose two non-null eigenvalues are the positive and negative square roots of:

$$\left[\frac{\alpha^A\beta^A\theta}{d_A(\alpha^A\psi+\sigma)} + \frac{\alpha^B\beta^B(1-\theta)}{d_B(\alpha^B\psi+\sigma)}\right]\left[\frac{\psi\sigma}{\rho(\omega+r)}\frac{F}{K}\right].$$

Therefore,

$$R_0^2 = \left[\frac{\alpha^A\beta^A\theta}{d_A(\alpha^A\psi+\sigma)} + \frac{\alpha^B\beta^B(1-\theta)}{d_B(\alpha^B\psi+\sigma)}\right]\left[\frac{\psi\sigma}{\rho(\omega+r)}f\right], \tag{S2.3}$$

where $f = F/K$ is the insect abundance per plant. In the case of a monoculture, we have

$$R_0^2 = \left[\frac{\alpha\beta}{d(\alpha\psi+\sigma)}\right]\left[\frac{\psi\sigma}{\rho(\omega+r)}f\right]. \tag{S2.4}$$

The basic reproduction number can be interpreted as follows:

$$R_0^2 = \begin{bmatrix} \text{Average success rate of} \\ \text{virus transmission per insect} \\ \text{on variety A} \end{bmatrix} + \begin{bmatrix} \text{Average success rate of} \\ \text{virus transmission per insect} \\ \text{on variety B} \end{bmatrix} \times \begin{bmatrix} \text{Relative rate of insect-mediated virus} \\ \text{transmission per plant} \\ \text{before infection is cleaned out} \end{bmatrix}$$

Where:

- The relative rate of insect-mediated virus transmission per plant before infection is cleaned out is the product of the **insect abundance per plant**, the **proportion of insects that successfully disperse to a new plant before dying or recovering**, **insect dispersal rate**, the **time between plant removal rounds** and the **time to virus cleaning out in insects**.

- The average success rate of virus transmission per insect on a plant variety is the product of the inoculation and the acquisition rates on the given variety adjusted for the overall contribution of insect activity (both acquisition and dispersal) to the detection and removal of infected plants.
