# Supplementary material for: Optimizing crop varietal mixtures for viral disease management: A case study on cassava virus epidemics
Source: PLoS Comput Biol. 2025 Sep 18;21(9):e1012842. doi: 10.1371/journal.pcbi.1012842 (PMC12469245; doi:10.1371/journal.pcbi.1012842)
Supplement: S3 Appendix — Generalisation to an n-variety model. Gives the general transition matrix, its inverse, and vector feeding proxies for arbitrary mixtures. (PDF) [file pcbi.1012842.s003.pdf]

S3 Appendix, Mixture of an arbitrary number of varieties

Let's consider n varieties $i \in \{1, \dots, n\}$ whose epidemic stages are S_i, L_i and I_i for all $i \in \{1, \dots, n\}$. Let's subdivide the viruliferous vector population V with regards to the plant variety on which vectors acquired the virus: $V^1 \equiv V^{I_1}, V^2 \equiv V^{I_2}, \dots, V^n \equiv V^{I_n}$. The model has $3n + n = 4n$ equations:

$$\begin{cases} \dot{S}_i = -\beta^i \dfrac{\Psi}{K} S_i \displaystyle\sum_{j=1}^{n} V^j + \rho d_i I_i \\ \dot{L}_i = \beta^i \dfrac{\Psi}{K} S_i \displaystyle\sum_{j=1}^{n} V^j - \gamma_i L_i \\ \dot{I}_i = \gamma_i L_i - \rho d_i I_i \\ \dot{V}^i = \alpha^i \left(I_i \dfrac{F}{K} - \dfrac{\Psi}{K} I_i \displaystyle\sum_{j=1}^{n} V^j \right) - (\omega + r) \left(1 + \alpha^i \dfrac{\Psi}{\sigma} \right) V^i, \qquad \text{for all } i \in \{1, \dots, n\}, \end{cases} \tag{S3.1}$$

where $\psi = \frac{\sigma}{\sigma+\omega+r}$ is the proportion of insects that successfully disperse to a new plant before dying or recovering, α^i, β^i, γ_i are respectively the rates of acquisition on variety i, inoculation on variety i, onset of disease in variety i, and d_i is the detection probability of infected plant of variety i. Indeed, let's name ρ^i_X the probability that a vector that acquired the virus on variety $i \in \{1, \dots, n\}$ is found feeding on a X plant, $X \in \{S_1, L_1, I_1, \dots, S_1, L_1, I_1, \dots, S_n, L_n, I_n\}$. We have for all $i \in \{1, \dots, n\}$:

$$\begin{cases} \dot{S}_i = -\beta^i \displaystyle\sum_{j=1}^{n} \rho^j_{S_i} V^j + \rho d_i I_i \\ \dot{L}_i = \beta^i \displaystyle\sum_{j=1}^{n} \rho^j_{S_i} V^j - \gamma_i L_i \\ \dot{I}_i = \gamma_i L_i - \rho d_i I_i \\ \dot{V}^i = \alpha^i \left(I_i \dfrac{F}{K} - \displaystyle\sum_{j=1}^{n} \rho^j_{I_i} V^j \right) - (r + \omega) V^i, \qquad \text{for all } i \in \{1, \dots, n\}. \end{cases} \tag{S3.2}$$

Let $\vec{\rho}^{\,i} = \left(\rho^i_X\right)_{X \in \{S_1, L_1, I_1, \dots, S_1, L_1, I_1, \dots, S_n, L_n, I_n\}}$, $i \in \{1, \dots, n\}$ be the i-th proxy vector. Similarly to the 2-varieties framework, we obtain the $3n$-dimensional transition square matrix :

$$M = (M_{ij})$$

with

$$M_{ii} = \begin{cases} -\left[\omega + \sigma(1 - \dfrac{S_i}{K}) + r\right], & \text{if } i = 1 \pmod 3 \\ -\left[\omega + \sigma(1 - \dfrac{L_i}{K}) + r\right], & \text{if } i = 2 \pmod 3 \\ -\left[\omega + \sigma(1 - \dfrac{I_i}{K}) + r\right], & \text{if } i = 0 \pmod 3). \end{cases}$$

And for $i \neq j$,

$$M_{ij} = \begin{cases} \sigma \dfrac{S_i}{K}, & \text{if } i = 1 \pmod 3 \\ \sigma \dfrac{L_i}{K}, & \text{if } i = 2 \pmod 3 \\ \sigma \dfrac{I_i}{K}, & \text{if } i = 0 \pmod 3 \end{cases}$$

The inverse matrix reads:

$$M^{-1} = \frac{1}{(\omega + r + \sigma)(\omega + r)} N,$$

where $N = (N_{ij})$ is the $3n$-dimensional square matrix defined by:

$$N_{ii} = \begin{cases} -\sigma \dfrac{S_i}{K} - r - \omega, & \text{if } i = 1 \pmod 3 \\ -\sigma \dfrac{L_i}{K} - r - \omega, & \text{if } i = 2 \pmod 3 \\ -\sigma \dfrac{I_i}{K} - r - \omega, & \text{if } i = 0 \pmod 3. \end{cases}$$

And for $i \neq j$,

$$N_{ij} = \begin{cases} -\sigma \dfrac{S_i}{K}, & \text{if } i = 1 \pmod 3 \\ -\sigma \dfrac{L_i}{K}, & \text{if } i = 2 \pmod 3 \\ -\sigma \dfrac{I_i}{K}, & \text{if } i = 0 \pmod 3 \end{cases}$$

Finally, for all $i \in \{1, \ldots, n\}$,

$$\vec{\rho}^{\,i} = M^{-1} \rho_0^{i^T},$$

with

$$\rho_0^{i^T} = (0, \ldots, 0, \underset{\substack{\uparrow \\ 3i}}{1}, 0, \ldots, 0),$$

from which

$$\vec{\rho}^{\,i} = \frac{1}{\omega + r + \sigma} \left(\sigma \frac{S_1}{K}, \sigma \frac{L_1}{K}, \sigma \frac{I_1}{K}, \ldots, \sigma \frac{S_i}{K}, \sigma \frac{L_i}{K}, \sigma \frac{I_i}{K} + \omega + r, \ldots, \sigma \frac{S_n}{K}, \sigma \frac{L_n}{K}, \sigma \frac{I_n}{K} \right).$$
