# Supplementary material for: Optimizing crop varietal mixtures for viral disease management: A case study on cassava virus epidemics
Source: PLoS Comput Biol. 2025 Sep 18;21(9):e1012842. doi: 10.1371/journal.pcbi.1012842 (PMC12469245; doi:10.1371/journal.pcbi.1012842)
Supplement: S4 Appendix — Example CropMix screenshots and sample outputs. Shows baseline and optimal mixture visualizations plus viruliferous vector dynamics. (PDF) [file pcbi.1012842.s004.pdf]

S4 Appendix, Graphical Outputs from CropMix

The graphical outputs generated by CropMix differ slightly from those presented in the main text. For instance, the app's output corresponding to CBSD, high insect pressure and monthly roguing is detailed as follows:

- Fig A illustrates the app's representation of the baseline mixture.
- Fig B depicts the app's visualization of the optimal mixture.

Beyond the epidemiological visualizations, the CropMix also includes displays of the viruliferous vector population dynamics.

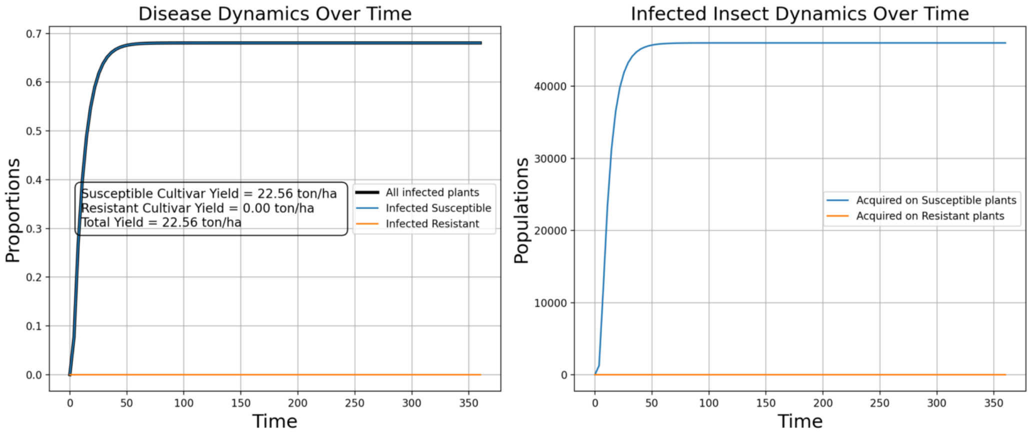

Fig A: App output example: monoculture of susceptible cassava for cassava brown streak disease high whitefly pressure and monthly roguing.

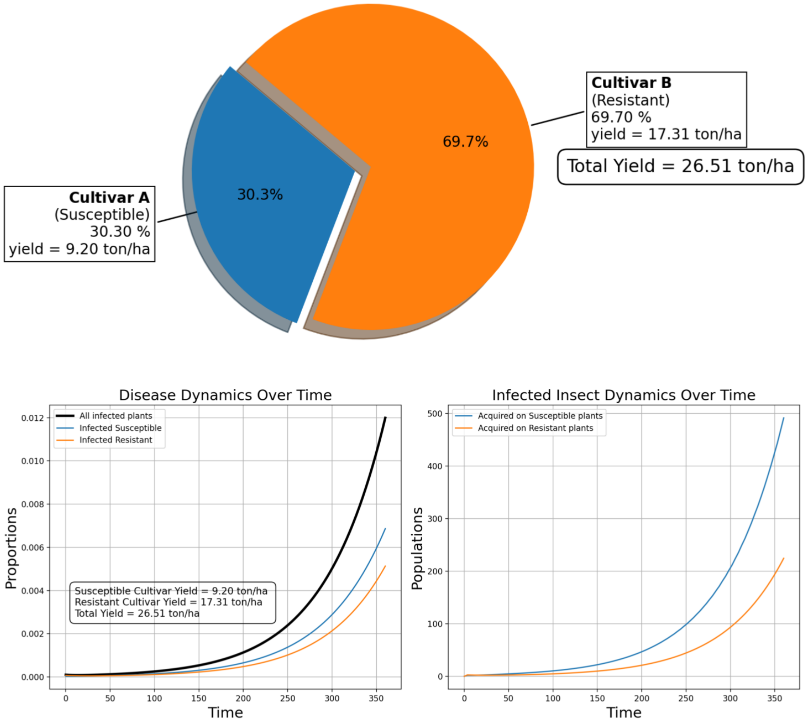

Fig B: App output example: a proportion of 69.7% of resistant plants is optimal for cassava brown streak disease, high whitefly pressure, and monthly roguing.
