# Supplementary material for: Optimizing crop varietal mixtures for viral disease management: A case study on cassava virus epidemics
Source: PLoS Comput Biol. 2025 Sep 18;21(9):e1012842. doi: 10.1371/journal.pcbi.1012842 (PMC12469245; doi:10.1371/journal.pcbi.1012842)
Supplement: S5 Appendix — The SUSC monoculture remains optimal across all roguing scenarios. (PDF) [file pcbi.1012842.s005.pdf]

S5 Appendix, Change in optimal *RES*-*SUSC* mixture with roguing under CMD

Decreasing the frequency of roguing (increasing $1/\rho$) tends to reduce the share of $SUSC$ in mixtures with RES. But, with CMD, even when starting from $1/\rho = 1$, a monoculture of $SUSC$ is consistently preferable to any mixture including RES: no mixture was able to protect $SUSC$ from CMD. the mixture (Fig A).

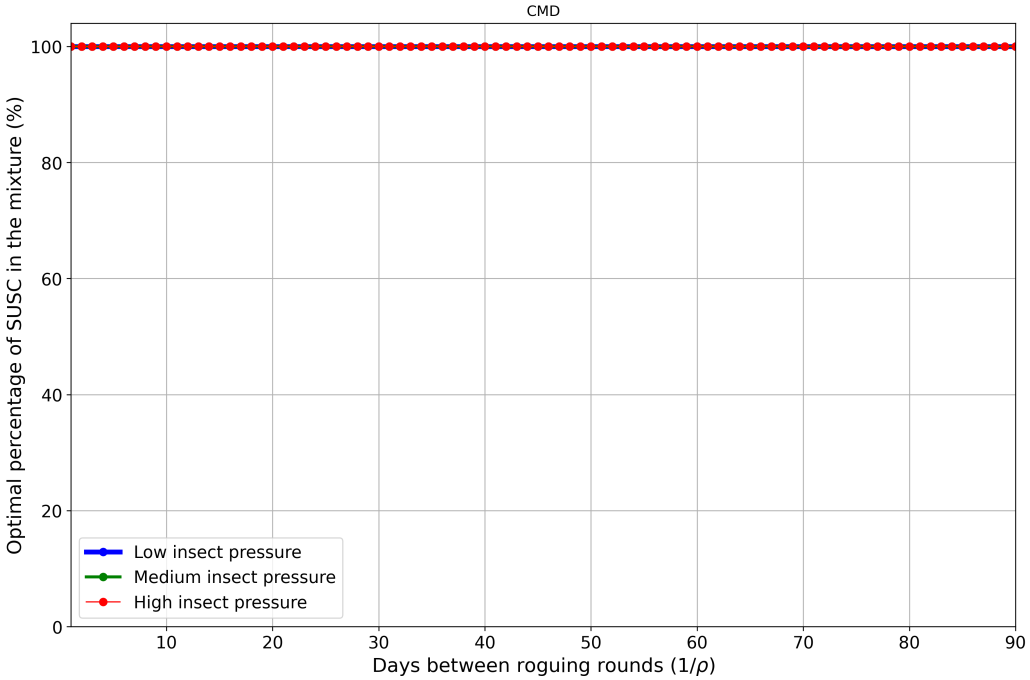

Fig A: Optimal proportion of $SUSC$ in a mixture with RES for CMD, plotted against the number of days between roguing events (i.e. $1/\rho$), under low, medium and high insect pressure. In all scenarios, the optimal $SUSC$ proportion remains at 100%, regardless of roguing frequency or vector burden, reflecting that no mixture with RES can protect susceptible cassava against CMD.

References

O. Diekmann, J. A. P. Heesterbeek, and M. G. Roberts. The construction of next-generation matrices for compartmental epidemic models. *Journal of The Royal Society Interface*, 7(47):873–885, 2009. ISSN 1742-5662. doi: 10.1098/rsif.2009.0386.

R. Donnelly, A. White, and M. Boots. The epidemiological feedbacks critical to the evolution of host immunity. *Journal of Evolutionary Biology*, 28(11):2042–2053, October 2015. ISSN 1420-9101. doi: 10.1111/jeb.12719.
